# Changes in Ca^2+^ Removal Can Mask the Effects of Geometry During IP_3_R Mediated Ca^2+^ Signals

**DOI:** 10.3389/fphys.2019.00964

**Published:** 2019-07-31

**Authors:** Estefanía Piegari, Cecilia Villarruel, Silvina Ponce Dawson

**Affiliations:** Departamento de Física FCEN-UBA and IFIBA (CONICET), Ciudad Universitaria, Buenos Aires, Argentina

**Keywords:** calcium signaling, spatiotemporal distribution, buffers, oocyte maturation, IP_3_R distribution

## Abstract

Calcium (Ca^2+^) signals are ubiquitous. Most intracellular Ca^2+^ signals involve the release of Ca^2+^ from the endoplasmic reticulum (ER) through Inositol 1,4,5-Trisphosphate Receptors (IP_3_Rs). The non-uniform spatial organization of IP_3_Rs and the fact that their individual openings are coupled *via* cytosolic Ca^2+^ are key factors for the variety of spatio-temporal distributions of the cytosolic [Ca^2+^] and the versatility of the signals. In this paper we combine experiments performed in untreated and in progesterone-treated *Xenopus laevis* oocytes and mathematical models to investigate how the interplay between *geometry* (the IP_3_R spatial distribution) and *dynamics* (the processes that characterize the release, transport, and removal of cytosolic Ca^2+^) affects the resulting signals. Signal propagation looks more continuous and spatially uniform in treated (mature) than in untreated (immature) oocytes. This could be due to the different underlying IP_3_R spatial distribution that has been observed in both cell types. The models, however, show that the rate of cytosolic Ca^2+^ removal, which is also different in both cell types, plays a key role affecting the coupling between Ca^2+^ release sites in such a way that the effect of the underlying IP_3_R spatial distribution can be modified.

## 1. Introduction

Calcium (Ca^2+^) signaling is involved in many physiological processes (Berridge et al., 1998). Most intracellular Ca^2+^ signals involve the release of Ca^2+^ from the endoplasmic reticulum (ER) through Inositol 1,4,5-Trisphosphate Receptors (IP_3_Rs). IP_3_Rs need to bind IP_3_ and Ca^2+^ on the cytosolic side to become open (Foskett et al., 2007). This means that individual IP_3_R openings are coupled *via* cytosolic Ca^2+^, a phenomenon that is known as *Calcium Induced Calcium Release* (CICR) (Callamaras et al., 1998). In most cell types, IP_3_Rs are organized in clusters. This leads to a wide range of release events depending on the location and quantity of channels which in turn depends on how strongly the different clusters are coupled through CICR. In particular, the use of slow Ca^2+^ buffers disrupts the inter-cluster coupling (Dargan and Parker, 2003; Piegari et al., 2015, 2018) limiting the spatial extent of the resulting signals. The variety of the signals therefore results from the interplay between *geometry* (the IP_3_R spatial distribution) and *dynamics* (the processes that characterize the release, transport, and removal of cytosolic Ca^2+^).

The main motivation of this paper is to study the relative role of geometry and dynamics on the resulting intracellular Ca^2+^ signal. To this end, we study the interplay between these two aspects experimentally and through modeling. Experimentally, we elicit, observe, and analyze IP_3_R-mediated Ca^2+^ signals in untreated (immature) and in progesterone-treated (mature) *Xenopus laevis* oocytes. It is known that the IP_3_R spatial distribution is different in these two situations (Terasaki et al., 2001; Machaca, 2004; Khaled, 2007) and it thus constitutes a natural setting where the effect of geometry can be studied. To analyze the changes in the resulting signal that are induced by variations in the spatial IP_3_R distribution we extend the model of Solovey and Dawson (2010); Lopez et al. (2012) to include the description of sequences of Ca^2+^ release events that occur in the same cell. In particular, we analyze the changes that arise in the signals when the same number of IP_3_Rs is distributed more or less uniformly over the cell.

The *fire-diffuse-fire* (*fdf*) model was introduced some years ago (Keizer et al., 1998; Dawson et al., 1999) to study theoretically to what extent the underlying non-uniform IP_3_R distribution is apparent on the propagating front of Ca^2+^ waves. This simple deterministic model is characterized by two dimensionless parameters: $\Gamma =\sigma /d^{3}/\left({\left[Ca\right]}_{T}-{\left[Ca\right]}_{b}\right)$ and β = *Dτ*/*d*^2^, where σ is the mean number of Ca^2+^ ions released per release site (i.e., IP_3_R cluster), τ is the mean duration during which a site releases Ca^2+^, *D* is the Ca^2+^ (effective) diffusion coefficient, *d* is the mean separation between release sites, [*Ca*]_*b*_ is the basal Ca^2+^ concentration, and [*Ca*]_*T*_ is a threshold Ca^2+^ concentration above which a site starts to release Ca^2+^. Γ determines whether the wave can propagate (if it is sufficiently large) or not. β rules whether the propagation is *saltatory* (β ≤ 1) or *continuous* which means that Ca^2+^ release from individual (localized) sites is apparent or is smeared out, respectively. In this paper signals that we observe experimentally in eggs and in oocytes have distinctive features that lead us to classify them as continuous and saltatory, respectively. Namely, the global waves that we observe in immature oocytes are preceded by localized signals (*puffs*) while those in eggs start, within the time resolution of the experiments, as soon as IP_3_ is released. In our experiments we also observe differences in the way that Ca^2+^ is cleared in the two cell types once the IP_3_ release is stopped which can be related to the existence of larger Ca^2+^ concentration gradients (i.e., less uniform Ca^2+^ distribution) in oocytes than in eggs. This distinction between saltatory and continuous signals is consistent with the differences between the waves elicited in immature oocytes (Yao et al., 1995) and the fertilization wave in eggs (Fontanilla and Nuccitelli, 1998). The study of the interplay between geometry and dynamics in Ca^2+^ signals can then shed light on what are the main factors involved in the change of propagation mode with maturation which might play a key role in guaranteeing that the Ca^2+^ fertilization wave can propagate without failure.

The mode of propagation (saltatory or continuous) is not the only difference between the waves elicited in immature eggs and the fertilization wave. The latter is slower (~ 9μ*m*/*s*) than the former (~ 20μ/*s*) (Dawson et al., 1999; Machaca, 2004). This might seem contradictory with the fact that saltatory waves can eventually fail to propagate. However, these aspects (slow but continuous propagation *vs* saltatory and fast propagation) could be accommodated within the *fdf* model in Dawson et al. (1999) assuming that σ/*d*^3^ was approximately equal in oocytes and eggs (~ 0.5), that Γ was only twice as large in eggs than in oocytes but that β was very different in both cases (0.08 in oocytes and 50 in eggs). It is interesting to notice that even if in these examples it is in principle “easier” to induce Ca^2+^ release from neighboring sites in the egg due to the larger Γ and *D* values, the wave propagates more slowly than in the oocyte. This shows that the mode of propagation (which determines whether *v* is proportional to *D*/*d* or to $\sqrt{D\tau }$) is key to set the wave speed. Now, there is another difference between the waves elicited experimentally in immature oocytes and the fertilization wave: while the IP_3_ distribution is approximately spatially uniform, within the observed region, this situation does not necessarily hold in the fertilization case. Namely, IP_3_ also starts to propagate from one end of the egg and even if IP_3_ is locally produced as the Ca^2+^ wave advances (Wagner et al., 2004), whether the IP_3_ concentration can be assumed to be uniform at the front of the Ca^2+^ wave depends on the relative speed with which the Ca^2+^ and the IP_3_ fronts advance. It is implicit in the comparison of Dawson et al. (1999) that IP_3_ travels fast enough so that it is uniform at the Ca^2+^ wave front. The idea that IP_3_ diffuses relatively fast in cells has been challenged recently in a work that shows that IP_3_ is also buffered in the cytosol (Dickinson et al., 2016). The factors that determine the speed of the fertilization wave should then be re-analyzed in view of these more recent observations.

In order to avoid uncertainties on the origin of the differences observed in different settings it is best to perform similar experiments in eggs and in immature oocytes and compare the observations. This is the approach that we follow for the experiments of this paper. This approach has been used before (Machaca, 2004; Sun et al., 2011). As done here, in these papers experiments were performed in eggs and oocytes with injected caged IP_3_ that was subsequently photo-released with UV light. In Machaca (2004), a sustained rise of the spatially averaged Ca^2+^ concentration that persisted in the region of IP_3_ uncaging was observed in the eggs, which is consistent with the 5–6 min plateau that is observed upon fertilization. Linescan images of this type of experiments, on the other hand, showed that Ca^2+^ signals tended to spread over a wider spatial region and lasted for a shorter time in eggs than in oocytes. The experiments performed in Machaca (2004) were elicited by a relatively brief UV pulse but of different duration in eggs and oocytes. In Sun et al. (2011), some signals were observed upon continuous photorelease of IP_3_, which also showed that they were more spatially localized in immature oocytes than in eggs (the authors talked about “signal coalescence” in the latter). In this paper the signal properties were contrasted against the structural changes that they observed occurred in the cells. Based on experimental observations with marked IP_3_Rs and ER, it was reported in Sun et al. (2011), that both the ER and the IP_3_R distribution were reticular in the immature oocyte while they showed a combination of “patches” and a reticular structure in eggs with Ca^2+^ release starting preferentially within the patches. The authors argued that IP_3_R-mediated Ca^2+^ release was *sensitized* in the patches and that this was key to determine the increased sensitization of this type of Ca^2+^ release process in eggs with respect to immature oocytes.

The modeling studies of Ullah et al. (2007) explained the larger Ca^2+^ release sensitization in eggs with respect to oocytes assuming that the affinity of IP_3_Rs for IP_3_ increased with maturation. The model of Sun et al. (2011), on the other hand, showed that increasing the IP_3_R density also “sensitized” the IP_3_R-mediated Ca^2+^ release. The effects of having different IP_3_R spatial distributions was analyzed in more detail in a subsequent modeling paper (Ullah et al., 2014). In this paper the authors found that they could reproduce the signals observed in oocytes by placing IP_3_R clusters with 20 IP_3_Rs each and an inter-cluster distance ~ 2.5μ*m*. In particular, they obtained abortive waves (i.e., waves that failed to propagate after a certain time). In order to obtain waves that propagated without failure at approximately the same speed as the Ca^2+^ fertilization wave, on the other hand, they used clusters with about 1,000 IP_3_Rs each and a larger (17.5μ*m*) inter-cluster distance. They found propagation failure, however, if the distance was increased to 20μ*m*, which seems to indicate that the propagation was saltatory for slightly smaller inter-cluster distances. To mimic the actual situation observed in Sun et al. (2011) some simulations of Ullah et al. (2014) were produced with two types of clusters: some with 980 IP_3_Rs each separated by 21μ*m* and others with 20 IP_3_Rs each separated by 3μ*m*. Since having smaller clusters inter-mixed with the large ones did not change the wave speed much the authors concluded that the latter were the main determinants of the propagation velocity.

Is the “larger” mean separation between the IP_3_R-patches what sets the speed of propagation of the wave in the egg? How could we have a larger mean separation between release sites in the egg when compared to the oocyte and, yet, have continuous propagation in the egg and saltatory in the oocyte? It is true that the transition from continuous to saltatory does not only depend on the mean separation of the IP_3_R clusters. In any case, the simulations of Ullah et al. (2014) do not show a very “continuous” front propagation in the case of the egg when it is assumed that a large fraction of the IP_3_Rs belong to a few clusters that are very separated among themselves while the rest are organized in more uniformly distributed smaller clusters. Furthermore, it seems as if the release of Ca^2+^ starts at various clusters before the front reaches them. These previous results show the need of keep on studying the interplay between geometry and dynamics on IP_3_R-mediated Ca^2+^ signals. This is the main goal of our paper. In particular, with our experiments we try to look for features that could indicate whether the resulting Ca^2+^ distribution is more or less spatially continuous in oocytes or eggs when they are subject to the same pattern of IP_3_ photo-release. With the numerical simulations, on the other hand, we try to determine the relative role of the spatial IP_3_R distribution and of some of the other factors that modulate the intracellular signals on the spatial distribution of the Ca^2+^ concentration and on propagation failure. In particular, we focus on how often an initial Ca^2+^ release eventually fails to lead to a more global solution, a feature that can be viewed as an “extremely saltatory" situation. We find that, even though the clusterization of IP_3_Rs is important to determine whether the propagation is saltatory or continuous, the rate at which Ca^2+^ is removed is key for this aspect as well. The numerical studies of our simple model show that by simply reducing the rate at which Ca^2+^ is removed the system changes from being excitable to being bistable and that this transition not only determines that the Ca^2+^ concentration can remain relatively large for a long time, but also has implications for the way the signal can propagate. From the combination of the experiments and the simulations we also find indications that the IP_3_R spatial distribution affects the “synchronicity” with which the IP_3_Rs go to the inhibited state, which, in turn, has an effect on how easy it is to propagate a subsequent signal or keep an elevated Ca^2+^ concentration. Different inhibition levels at more or less densely packed clusters were also observed in the simulations of Ullah et al. (2007).

## 2. Materials and Methods

### 2.1. Oocyte Preparation

Adult female *X. laevis* Nasco, Fort Atkinson, WI, USA were maintained in a room with controlled temperature (18°C) and a 12-h light-dark cycle. Each frog was kept in an individual tank with filtered water and was fed twice a week. Frogs were anesthetized for surgery by immersion in 0.3% tricaine (MS222) and oocytes were removed and prepared as previously described in Goldman et al. (2017). All procedures were carried out in accordance with the rules defined by the local Council for the Correct Use and Care of Laboratory Animals, which complies with the EU Directive 2010/63/EU. The protocol was approved by CICUAL.

Experiments were performed in both immature and mature *X. laevis* oocytes previously treated with collagenase. Oocytes were loaded by intracellular microinjection with different compounds. The calcium dye Fluo-4 dextran high affinity (*K*_*d*_= 772 nM) was used to probe cytosolic [Ca^2+^]. Caged IP_3_ (D-Myo-Inositol 1,4,5-Triphosphate,P4(5)-(1-(2-Nitrophenyl)ethyl) Ester) was used to induce IP_3_Rs opening. Final intracellular concentrations of the different compounds were calculated assuming a 1μl cytosolic volume. Final intracellular concentration of IP_3_ and Fluo-4 were 9 and 36 μM, respectively, in all the experiments. Fluo-4 and IP_3_ were from Molecular Probes Inc. Recordings were made at room temperature.

Oocytes were artificially matured by incubating them in progesterone with a 2.5μ*g*/*ml* concentration at 18°C during 12–16 h. Eggs with white dots in the animal hemisphere were chosen since this indicates germinal vesicle break down (GVBD). Experiments were performed between 3 and 4 h after the white's dot appearance. At this time it is supposed that the egg is at metaphase II of meiosis and that maturation is complete (Gallo et al., 1995; Sun and Machaca, 2004).

The total number of oocytes and eggs where signals were observed is 8 and 5, respectively. In 6 oocytes and 2 eggs the regions were fixed and varied in the rest of them.

### 2.2. Confocal Microscopy

Confocal imaging was performed using a spectral confocal scanning microscope Olympus FluoView1000 that has a spectral scan unit connected to an inverted microscope IX81. Fluo-4 was excited with the 488 nm line of a multiline Argon laser focused on the oocyte with a 60× oil immersion objective (NA 1.35). The emitted fluorescence was detected in the 500–600 nm range with PMT detectors. Images were acquired in the frame mode over regions of 250 × 250 pixels (207 × 207μ*m*) with a 4μ*s* time per pixel and 0.56*s* by frame. The Ca^2+^ signals were elicited photolyzing the caged IP_3_ with the UV part of the spectrum of a mercury lamp that comes with the microscope using the modification introduced in Sigaut et al. (2011). In all the experiments, two UV flashes of 1.68 s duration were applied separated by different time intervals. The first UV flash was always applied after the image acquisition had started.

### 2.3. Image Analysis and Event Characterization

The experiments gave sequences of frames of 250 × 250 pixels each. Detector noise was small and, hence, it was not necessary to filter the spot noise (van Wijk, 1991). To characterize differences in the propagation of the signals between oocytes and eggs we divided the frames in 25 (spatial) subregions and computed the mean fluorescence, ${\bar{F}}_{k}\left(t\right)$, for each subregion as a function of time, *t*, as:

(1) $$\begin{array}{l} {\bar{F}}_{k}\left(t\right)=\frac{1}{N_{T_{k}}}\overset{N_{k}}{\underset{i=1}{\sum }}\overset{N_{k}}{\underset{j=1}{\sum }}F\left(i_{k},j_{k},t\right),\;k=1,\ldots ,25, \end{array}$$

where the sum is over the pixels (*i*_*k*_, *j*_*k*_) of the *k*-th subregion and *N*_*T*_*k*__ = *N*_*k*_ · *N*_*k*_, with *N*_*k*_ = 50 ∀*k* ∈ [1, 25], is the total number of pixels of the *k*-th subregion.

We also computed the mean fluorescence over the whole image, *F*_*m*_, and the standard deviation, σ_*F*_, as:

(2) $$\begin{array}{lll} F_{m}\left(t\right) & = & \frac{1}{n}\underset{k/{\bar{F}}_{b_{k}}>F_{min}}{\sum }{\bar{F}}_{k}\left(t\right), \end{array}$$

(3) $$\begin{array}{lll} \sigma_{F}\left(t\right) & = & {\left(\frac{1}{n-1}\underset{k/{\bar{F}}_{b_{k}}>F_{min}}{\sum }{\left({\bar{F}}_{k}\left(t\right)-F_{m}\left(t\right)\right)}^{2}\right)}^{1/2}, \end{array}$$

where the sums ran over the *n* subregions with “basal” fluorescence, ${\bar{F}}_{b_{k}}$, above a minimum value, *F*_*min*_. We computed the basal fluorescence, ${\bar{F}}_{b_{k}}$, of the *k*-th region as the time average of ${\bar{F}}_{k}\left(t\right)$ before the first UV flash. The minimum value, *F*_*min*_, was computed as $F_{min}=\langle {\bar{F}}_{b}\rangle -1.5\sigma_{{\bar{F}}_{b}}$ with $\langle {\bar{F}}_{b}\rangle$ and $\sigma_{{\bar{F}}_{b}}$ the mean and standard deviation of the time average (before the UV flash) of ${\bar{F}}_{k}$ over the 25 subregions of the frame.

Both ${\bar{F}}_{k}$ and *F*_*m*_ give information on the fluorescence time course and, therefore, on the Ca^2+^ concentration in the corresponding region. We characterized the characteristic times of growth and decay for both types of fluorescence traces [which we will call, generically, *F*(*t*)] in the following way. We computed the rise time as $t_{r}=\frac{F_{M}+F_{b}}{2\left(F_{M}-F_{b}\right)}\cdot \Delta t$ with *F*_*M*_ the maximum value of *F*(*t*), *F*_*b*_ the value of *F*(*t*) immediately before the UV flash and Δ*t* the time elapsed between the initiation of the flash and the occurrence of the fluorescence maximum. The decay time was obtained by fitting *F*(*t*) over the time interval that went from the first frame-time after the UV flash had been turned off (*t*_*o*_) to the time at which *F*(*t*) reached the value 1.5 × *F*_*b*_. We tried three types of fittings:

(4) $$\begin{array}{l} F=A\cdot e^{-\left(t-t_{o}\right)/t_{df}}+B\cdot e^{-\left(t-t_{o}\right)/t_{ds}}, \end{array}$$

(5) $$\begin{array}{l} F=C\cdot e^{-\left(t-t_{o}\right)/t_{dm}}, \end{array}$$

(6) $$\begin{array}{l} F=F_{0}\cdot \left(1-\left(t-t_{o}\right)/t_{dl}\right), \end{array}$$

i.e., bi-exponential, mono-exponential, and linear, respectively. For each case we obtained the characteristic decay times, *t*_*df*_ and *t*_*ds*_ the fast and slow decay times obtained after fitting with Equation (4), *t*_*dm*_ the decay time of the monoexponential fit (Equation 5) and *t*_*dl*_ the decay time obtained when fitting with Equation (6).

### 2.4. Numerical Simulations

**Figure 1:**
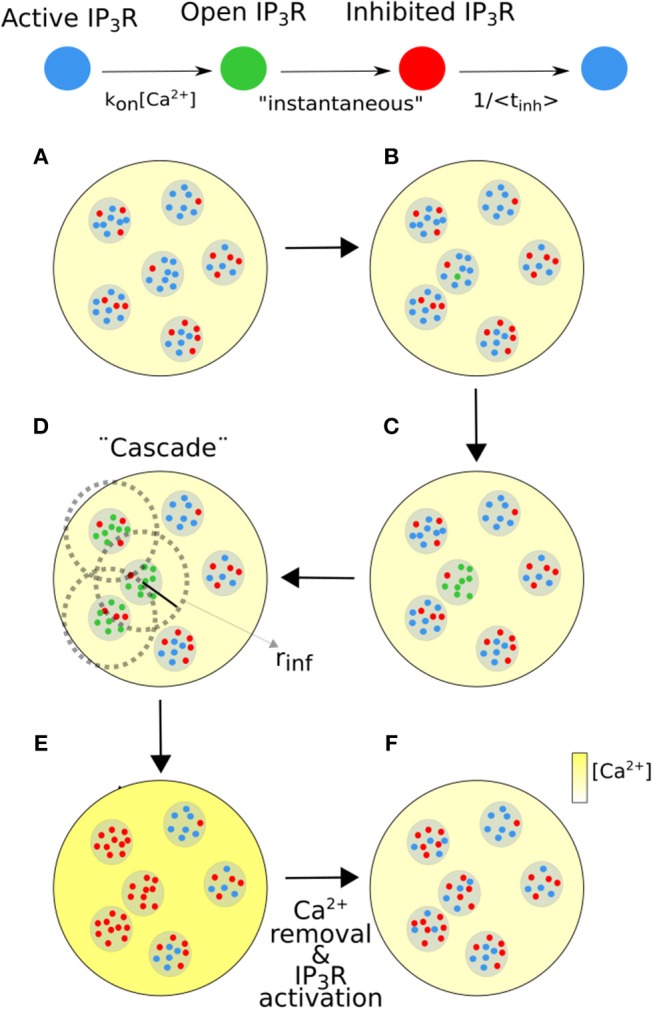
Scheme of the numerical simulation steps. **(A,B)** An active IP_3_R (blue dot) becomes open with a probability per unit time that depends on [Ca^2+^]. **(C)** An open IP_3_R (green dot) induces the opening of all active IP_3_Rs in its cluster. **(D)** A cluster with *N*_*o*_ open IP_3_Rs induces the opening of all active IP_3_Rs inside clusters that are within a distance, *r*_*inf*_, from it (dotted circle). *r*_*inf*_ is an increasing function of *N*_*o*_ and (cytosolic) [Ca^2+^] (yellow color bar). This “cascade” is instantaneous. **(E)** [Ca^2+^] increases and the open IP_3_Rs become inhibited (red dots). **(F)** When Ca^2+^ is removed the inhibited IP_3_Rs can become active.

Numerical simulations were performed using a modified version of the model introduced in Lopez et al. (2012) in which all IP_3_Rs were initially closed. Briefly, the simulation domain was a π*R*^2^ circular region with *R* = 10μ*m*. *N* IP_3_R clusters were initially placed at random in the domain choosing their positions with uniform distribution over the circle. For each simulation, the number, *N*, was chosen from a Poisson distribution with mean, $\lambda_{N}={\left(2R\right)}^{2}/dm^{2}$. This guaranteed that the mean separation between the clusters was *dm*. The number of IP_3_Rs in each cluster was chosen from a Poisson distribution with mean, *N*_*I*_*P*__3_*R*_, at the beginning of the simulation. All IP_3_Rs were assumed to be IP_3_-bound and initially active. In the model an *event* is a sequence (or *cascade*) of IP_3_R openings coupled via CICR. The time propagation of the signal is not described, it is instantaneous (Solovey and Dawson, 2010). An scheme of the numerical simulations steps is shown in Figure 1. An event is triggered when one active IP_3_R becomes open (Figures 1A,B). When this occurs, all the active IP_3_Rs of the same cluster become open as well (Figure 1C). These *n*_*o*_ open IP_3_Rs induce the opening of all active IP_3_Rs in clusters within a distance, *d*, of the first one that depends on the level of Ca^2+^ in the medium, [*Ca*_*i*_], before the beginning of the event and on *n*_*o*_ according to: 0.0414*n*_*o*_μ*Mμm*/*d* + [*Ca*_*i*_] ≥ 0.0414μ*Mμm*/*r*_*inf*_ + 0.1μ*M*, where [*Ca*_*i*_] is the cytosolic Ca^2+^ concentration immediately before the event and *r*_*inf*_ = 0.25μ*m* (Lopez et al., 2012) (see Figure 1D). This process is repeated until no more IP_3_Rs fulfill the CICR condition. All IP_3_Rs that participate of the event become inactive immediately afterwards (Figure 1E). An inactivation time is chosen for each of them from an exponential distribution of mean, *t*_*inh*_ = 2.5*s* (Fraiman et al., 2006). Once its inactivation time has elapsed the corresponding IP_3_R becomes active again (Figure 1F). We characterize the event in terms of the total number of IP_3_Rs that become open, *N*_*o*_. Between events we assume that [Ca^2+^] starts from a high (homogeneous) level that depends on the latest *N*_*o*_ and that subsequently decreases exponentially with timescale 1/δ_*Ca*_ (Lopez et al., 2012). Time is advanced with time step *dt* = 0.05*s* between events. A new event starts at a cluster with probability per unit time that depends on the number of active IP_3_Rs at the cluster and on the current level of [Ca^2+^]. In particular, the probability per unit time that an active IP_3_R in a cluster with *N*_*act*_ active IP_3_Rs becomes open is $0.225s^{-1}\cdot N_{act}\cdot \left[Ca^{2+}\right]/{\left[Ca^{2+}\right]}_{basal}$. This probability per unit of time matches the one in Fraiman et al. (2006) when Ca^2+^ is at basal concentration. The simulation starts at *t* = 0 when all IP_3_Rs are active and the [Ca^2+^] is ${\left[Ca^{2+}\right]}_{basal}=0.1\mu M$.

In order to illustrate how the signal associated to an event with the number and location of the participating IP_3_Rs prescribed by the simulations looks like, we estimated the [Ca^2+^] distribution as:

(7) $$\begin{array}{l} \left[Ca^{2+}\right]={\left[Ca^{2+}\right]}_{prev}+\overset{N_{c}}{\underset{i}{\sum }}N_{oi}\cdot A\cdot \exp\left(-\left({\left(x-x_{i}\right)}^{2}+{\left(y-y_{i}\right)}^{2}\right)/\left(2\sigma^{2}\right)\right), \end{array}$$

where ${\left[Ca^{2+}\right]}_{prev}$ is the Ca^2+^ concentration immediately before the signal occurred; the subscript, *i*, identifies the *i*-th cluster whose position is (*x*_*i*_, *y*_*i*_); *N*_*oi*_ is the number of channels of that cluster that participated of the signal and the function that multiplies *N*_*oi*_ is a Gaussian approximation of the [Ca^2+^] contribution of a 0.1*pA* Ca^2+^ point source. The amplitude of this Gaussian is $A=9.88\mu M\mu m^{3}/\left(D\cdot dr\right)$, where *D* = 100μ*m*^2^/*s* is the Ca^2+^ diffusion coefficient, *dr* determines the “coarse-graining” with the [Ca^2+^] contribution of the point source is computed (*dr* = 0.16μ*m*) and σ^2^ = 2μ*m*^2^ gives the width of the Gaussian.

## 3. Results

Here we present the results of experiments performed in immature oocytes of *X. laevis* and in oocytes of the same species maturated with progesterone (eggs) as described in section 2. We also show the results of numerical simulations of the model described in Materials and Methods. The aim of these studies is to analyze the differences and similarities between the IP_3_R-mediated signals evoked in eggs and in immature oocytes and to establish the factors that are key to determine their differences.

### 3.1. Experiments

**Figure 2:**
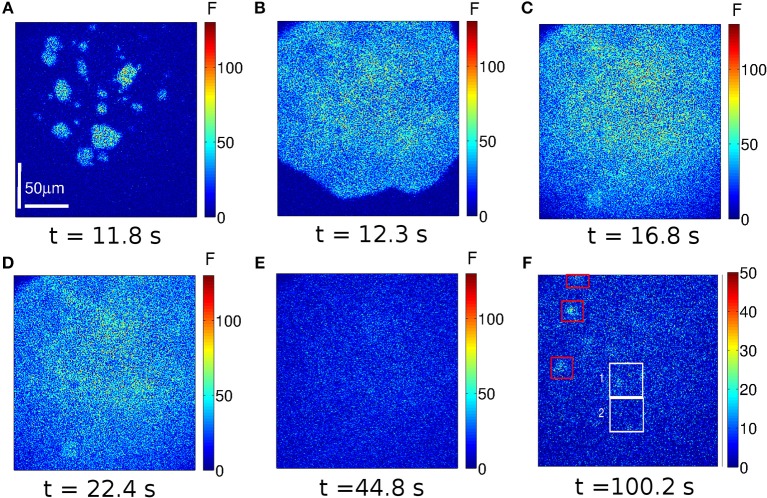
Example of a Ca^2+^ signal evoked in an immature oocyte. Frames of 250 × 250 pixels acquired at the indicated times. Warmer colors correspond to increasing fluorescence values in arbitrary units (*a*.*u*.). The UV illumination (used to uncage the IP_3_) was on between *t* = 11.2 s and *t* = 28 s and between *t* = 173.6 s and *t* = 190.4 s. At *t* ~ 11.8 s various localized Ca^2+^ elevations (*spotlights*) are apparent **(A)**. They eventually lead to a wave **(B)** that propagates throughout the observed region. The maximum fluorescence level is reached at *t* ~ 16.8*s* **(C)**. A frame obtained slightly after shows a lower fluorescence level **(D)**. After the (first) UV flash is turned off (*t* = 28 s) the fluorescence decays rapidly until it reaches the basal level by *t* = 44.8 s **(E)**. Various localized signals (*puffs*) arise in between the two UV flashes as illustrated in **(F)**. The white boxes indicate two regions analyzed in **Figure 4**.

**Figure 3:**
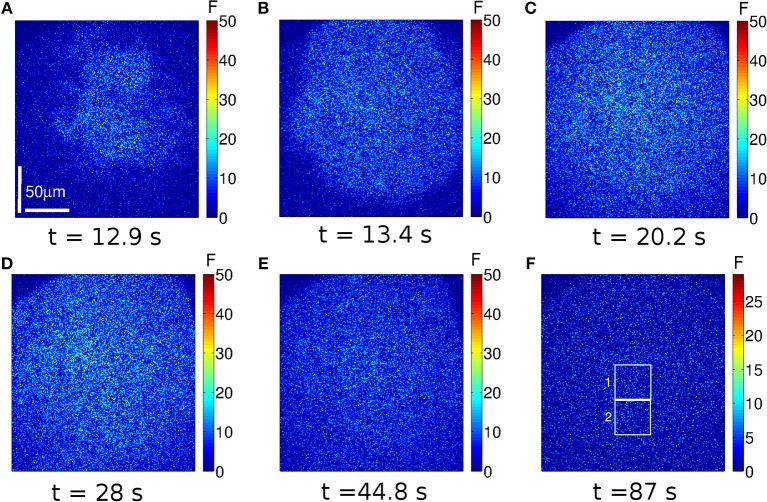
Example of a Ca^2+^ signal evoked in an egg. Similar to Figure 2, but for an experiment performed in an egg. In this case the UV illumination was on between *t* = 11.2 s and *t* = 28 s and between *t* = 112 s and *t* = 128.8 s. At *t* ~ 12.9 s the Ca^2+^ release becomes apparent **(A)**. The Ca^2+^ distribution is more spatially uniform than in the oocyte. The wave propagates **(B,C)** and the fluorescence keeps on increasing while the UV light is on **(D)**. Approximately 16 s after the UV flash is turned off (*t* = 44.8 s), the fluorescence has not reached the basal level yet **(E)**. Ca^2+^ puffs are not observed in between the two UV flashes **(F)**. The white boxes indicate two regions analyzed in Figure 4.

The images we show in Figures 2, 3 are representative of the behavior observed in (immature) oocytes and in eggs, respectively. Frames acquired at six different times (indicated in the figure) are shown. The complete videos can be seen in Supplementary Videos 1, 2. The size of the images is 207 × 207μ*m*, the scale is indicated with a white line. The color bar represents the fluorescence value, *F*, at each pixel of the image in arbitrary units. In these two examples the UV light used to uncage the IP_3_ was turned on at *t* = 11.2 s and turned off at *t* = 28 s and then turned back on at *t* = 173.6 s and off at *t* = 190.4 s in the case of the oocyte (Figure 2) and on at *t* = 112 s and off at *t* = 128.8 s in the case of the egg (Figure 3).

In the oocyte we observe that various localized signals are elicited almost simultaneously in different regions ~ 0.6 s after the UV flash is turned on (Figure 2A). A spatially more uniform signal is generated from these “spotlights” (Figure 2B) that eventually encompasses all of the observed region. The Ca^2+^ elevation remains high until *t* ~ 16.8 s (Figure 2C) after which it starts to decay slightly (e.g., Figure 2D). Once the UV flash is turned off there is an abrupt decay and a subsequent slower decay of the fluorescence to the basal level (Figure 2E). After the first UV flash is turned off but before the second one is turned on several puffs turn on and off in different regions of the cell. The frame at *t* = 100.2 s shows several puffs in different points of the image, albeit at a lower fluorescence level (Figure 2F) than in the case of the wave (Figures 2A–D). The same behavior is observed after the second UV pulse.

In the case of the egg, the fluorescence starts to increase at *t* = 12.9 s (~ 1.6 s after the initiation of the UV flash, see Figure 3A) in a more uniform way over the observed region than in the oocyte. Clearly visible localized signals are not identified in this case. The signal propagates (Figures 3B,C) and the level of fluorescence keeps on increasing until the UV light is turned off (*t* = 28 s). Differently from the case of the oocyte, by *t* ~ 44.8 s the egg has not reached the basal level yet (Figure 3D). Furthermore, even if the time elapsed between the end of the first UV flash and the beginning of the second is shorter for the egg than for the oocyte, localized signals are not observed in the egg in between these two times (Figure 3E). Another difference between Figures 2, 3 is the maximum value of the fluorescence that is attained during the signal: it is above 100 *a*.*u*. in Figure 2 and it never exceeds 50 *a*.*u*. in Figure 3.

**Figure 4:**
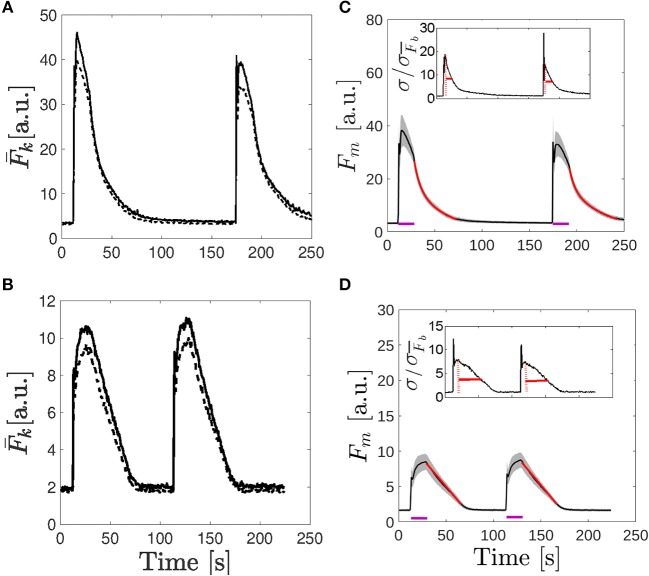
Comparison of the fluorescence time course obtained in the examples of Figures 2, 3. (A,B) Time course of the fluorescence, ${\bar{F}}_{k}$, averaged over the two 50 × 50 pixel subregions depicted in Figure 2F (A) and Figure 3F (B). In both cases, the solid line corresponds to *k* = 1 and the dashed one to *k* = 2. **(C,D)** Mean fluorescence, *F*_*m*_ given by Equation (1) (black curve), and region around determined by the standard deviation, *F*_*m*_ ± σ_*F*_ with σ_*F*_ given by Equation (3) (shaded area) as functions of time for the immature oocyte **(C)** and for the egg **(D)**. Fits to the traces after the UV flash was turned off are shown in red [bi-exponential fit given by Equation 4 in **(C)** and linear fit given by Equation 6 in **(D)**]. The purple lines indicate the times during which the UV pulses were on. In the insets the ratio, $\sigma_{F}\left(t\right)/\sigma_{{\bar{F}}_{b}}$, with $\sigma_{{\bar{F}}_{b}}$, the deviation before the first UV flash was delivered, is plotted. The maximum ratio is indicated with a dotted red line and the solid red line indicates the time it takes for the ratio to fall by half.

To study if spatial inhomogeneities exist within the observed region, we computed the fluorescence, ${\bar{F}}_{k}\left(t\right)$, given by Equation (1), for the two subregions (region 1 and 2) delimited by the white boxes of Figures 2F, 3F. We plot the traces obtained in Figures 4A,B for the oocyte and the egg, respectively. As described in section 2, we computed the rise time of the four functions, ${\bar{F}}_{k}\left(t\right)$, for each of the two UV flashes. We also fitted the time dependence of the four functions, ${\bar{F}}_{k}\left(t\right)$, after each of the UV flash had been turned off to derive decay times. We quote here the timescales obtained for the oocyte using Equation (4) to infer the decay times and those obtained for the egg using Equation (6). For region 1 of the oocyte we obtained *t*_*r*_ = 2.3 s, *t*_*df*_ = 5.7 s, and *t*_*ds*_ = 36.4 s for the first UV pulse and *t*_*r*_ = 3.0 s, *t*_*df*_ = 5.9 s, and *t*_*ds*_ = 44.9 s for the second pulse. In region 2 of the oocyte we obtained *t*_*r*_ = 2.0 s, *t*_*df*_ = 5.8 s, and *t*_*ds*_ = 30.0 s for the first pulse and *t*_*r*_ = 2.0 s, *t*_*df*_ = 5.2 s, and *t*_*ds*_ = 37.3 s for the second pulse. In region 1 of the egg we obtained: *t*_*r*_ = 9.8 s and *t*_*dl*_ = 53.5 s for the first pulse and *t*_*r*_ = 10.9 s and *t*_*dl*_ = 51.7 s for the second pulse. In region 2 of the egg we obtained: *t*_*r*_ = 9.8 s and *t*_*dl*_ = 52.0 s for the first pulse and *t*_*r*_ = 10.9 s and *t*_*dl*_ = 52.6 s for the second pulse.

To further characterize the dynamics of the signals of Figures 2, 3 we computed *F*_*m*_(*t*) and σ_*F*_(*t*) as defined in Equations (2) and (3). The number of subregions with similar basal fluorescence levels was *n* = 20 in the case of the oocyte [${\bar{F}}_{b_{k}}\in \left(2.6,3.8\right)a.u.$] and *n* = 21 [${\bar{F}}_{b_{k}}\in \left(1.4,2\right)a.u.$] in the case of the egg. We show in Figures 4C,D, the results obtained for the oocyte and the egg, respectively. In these figures the black solid line corresponds to *F*_*m*_ and the gray shaded area covers the region *F*_*m*_ ± σ_*F*_. These figures illustrate how the cells respond to the two 16.8 s long UV flashes, the first one that started at *t* = 11.2 s and the second one that started *t* = 173.6 s for the oocyte and at *t* = 112 s for the egg, as indicated by the purple horizontal lines in the figures. We derived the rise time of *F*_*m*_ and fitted its decaying part once the UV flash was turned off using Equation (4) for the oocyte and Equation (6) for the egg as explained in section 2. The fitting curves are shown in red in Figures 4C,D. The characteristic times that we obtained in the case of the oocyte were: *t*_*r*_ = 2.0 s, *t*_*df*_ = 5.7 s, and *t*_*ds*_ = 34.2 s for the first pulse and *t*_*r*_ = 3.0 s, *t*_*df*_ = 5.8 s, and *t*_*ds*_ = 41.2 s for the second one. In the case of the egg we obtained: *t*_*r*_ = 11.3 s and *t*_*dl*_ = 52.6 s for the first pulse and *t*_*r*_ = 12.0 s and *t*_*dl*_ = 52.6 s for the second pulse. In the insets of Figures 4C,D it is shown the ratio, $\sigma_{F}\left(t\right)/\sigma_{{\bar{F}}_{b}}$, with $\sigma_{{\bar{F}}_{b}}$, the deviation before the first UV flash was delivered. The maximum ratio is indicated with a dotted red line and the solid red line indicates the time it takes for the ratio to fall by half.

**Figure 5:**
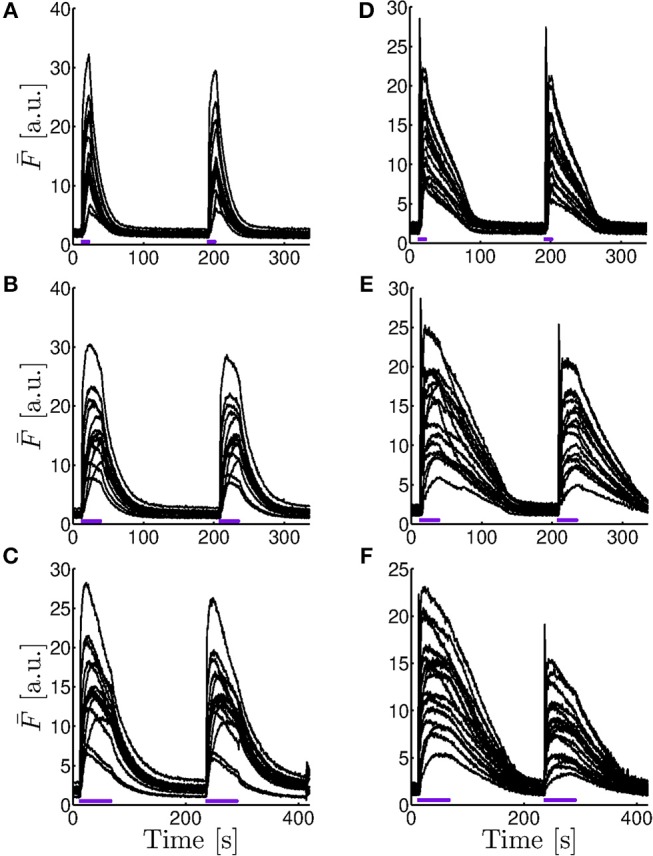
Comparison of the fluorescence time course obtained in experiments performed for different UV pulses. Fluorescence averaged over subregions with similar basal fluorescence levels (${\bar{F}}_{k}$, Equation 1) as a function of time for experiments performed in an immature oocyte **(A–C)** and in an egg **(D–F)** in which two UV pulses of 11.2 s **(A,D)**, 28 s **(B,E)**, and 56 s **(C,F)** were applied to photo-release the caged IP_3_. The time elapsed between the end of the first flash and the beginning of the second was always s. The number of curves and, equivalently, of subregions, is *n* = 15 in the oocyte **(A–C)** and *n* = 16 in the egg **(D–F)**.

In order to study whether the behaviors observed in Figures 2–4 persist under other conditions, particularly, of IP_3_ uncaging, we repeated the experiments for different durations of the UV flash both in oocytes and in eggs. Two UV flashes of the same intensity were applied in all cases. While the time elapsed between the end of the first flash and the beginning of the second was always 168 s, the duration of the flashes was different depending on the experiment. We show in Figure 5 the time course of the fluorescence, ${\bar{F}}_{k}\left(t\right)$ (Equation 1), obtained for the various 41.4 × 41.4 μm regions with similar basal fluorescence levels in which we subdivided the frames for the different experiments. The figures to the left correspond to experiments performed in an oocyte and those to the right to experiments performed in an egg. The experiments were repeated in other oocytes and eggs obtaining qualitatively similar results (data not shown). The duration of the flashes increases from top to bottom: it is 11.2 s in Figures 5A,B, 28 s in Figures 5C,D, and 56 s in Figures 5E,F.

As in the examples of Figure 4, we also tried different fits (Equations 4–6) to characterize the decay of the fluorescence, ${\bar{F}}_{k}\left(t\right)$ (Equation 1), once the UV flash was turned off, for all the subregions in which we sub-divided the images. As explained in section 2, for each experiment we identified the *n* subregions with similar basal fluorescence levels and computed ${\bar{F}}_{k}\left(t\right)$ for each of them. We then fitted the time course, immediately after each UV flash was turned off, of the *n* functions, ${\bar{F}}_{k}\left(t\right)$, obtained for each experiment. For the fittings, we used Equations (4) and (5) in the case of the experiments performed in oocytes and Equations (5) and (6) in the case of the those performed in eggs. For each case we obtained the characteristic decay times, *t*_*df*_ and *t*_*ds*_ the fast and slow decay times obtained after fitting with Equation (4), *t*_*dm*_ the decay time of the monoexponential fit (Equation 5) and *t*_*dl*_ the decay time obtained when fitting with Equation (6). We then computed the mean and standard deviation of the *n* characteristic times derived for each experiment and each pulse. In Table 1 we list the results obtained for the experiment of Figure 2 and for all the experiments performed in oocytes of Figure 5. We list in Table 2 those obtained for the experiment of Figure 3 and for all the experiments performed in eggs of Figure 5. Cases for which we do not report results are those in which the fitting procedure did not converge.

**Table 1:** Mean, standard deviation, and coefficient of determination of the characteristic times derived from the monoexponential, *t*_*dm*_ (Equation 5) and bi-exponential, *t*_*df*_ and *t*_*ds*_ (Equation 4) fits of the decaying part of the fluorescence, ${\bar{F}}_{k}\left(t\right)$ (Equation 1), observed in oocytes for different UV flash durations.

Pulse number and UV flash duration [s]	Mono-exponential fit			Bi-exponential fit				
	*t*_dm_[*s*]	*SD*[*s*]	*R*^2^	*t*_df_[*s*]	*SD*[*s*]	*t*_ds_[*s*]	*SD*[*s*]	*R*^2^
#1, 11.2	20.4	6.5	0.9942	7.3	5.2	23.8	8.9	0.9976
#1, 16.8	—			5.5	0.7	34.5	6.4	0.9986
#1, 28	27	2.5	0.9922	13.8	7.8	49.8	32.4	0.9985
#1, 56	52.4	5.7	0.9779	20.1	4.9	133.7	64.7	0.9972
#2, 11.2	20.6	6.8	0.9903	—				
#2, 16.8	—			5.1	1.2	41.7	8.5	0.9970
#2, 28	28.8	2.5	0.9909	11.5	7.8	37.2	14.5	0.9981
#2, 56	71.1	9.8	0.9854	—				

*The number of analyzed regions was n = 20 for the 16.8 s flash duration and n = 15 for the rest. See main text for other details*.

**Table 2:** Similar to Table 1, but for the parameters derived from the linear (Equation 6) and the mono-exponential (Equation 5) fits of the decaying part of the fluorescence, ${\bar{F}}_{k}\left(t\right)$ (Equation 1), observed in eggs.

Pulse number and UV flash duration [s]	Linear fit			Mono-exponential fit		
	*t_dl_*[*s*]	*SD*[*s*]	*R*^2^	*t*_dm_[*s*]	*SD*[*s*]	*R*^2^
#1, 11.2	82.6	2.6	0.9899	53.6	4.3	0.9684
#1, 16.8	52.2	1.3	0.9916	34	1.3	0.9899
#1, 28	121.4	9.5	0.9868	75.8	11	0.9693
#1, 56	145.8	5.3	0.9830	83	5.8	0.9846
#2, 11.2	79.9	6.1	0.9893	51.6	7.8	0.982
#2, 16.8	52.3	2	0.992	33.9	1.8	0.9897
#2, 28	116.3	9.5	0.9847	68.8	9.6	0.9897
#2, 56	142.8	10.6	0.9488	83.3	9.9	0.9854

*The number of analyzed regions was n = 21 for the 16.8 s flash duration and n = 16 for the rest*.

### 3.2. Numerical Simulations

In this section we present the results of numerical simulations of the model described in section 2.4. We list in Table 3 the values of the parameters that were varied between simulations: the mean separation between clusters, *dm*; the mean number of clusters, λ_*N*_; the mean number of IP_3_-bounded IP_3_Rs in each cluster, *N*_*IP*3*R*_; and the cytosolic Ca^2+^ removal rate, δ_*Ca*_. The aim of the simulations is to study to what extent a more or less uniform IP_3_R spatial distribution impacts on the resulting signal. Thus, we chose some sets of parameters for which the total number of IP_3_Rs, *N*_*T*_ (also shown in Table 3), is approximately the same but the way the channels are spatially distributed is different. Other simulations are aimed at studying how the results vary depending on the rate of Ca^2+^ removal. Differently from the experiments, the model assumes that [IP_3_] is constant. It does not describe the time during which the signals propagate either. It allows to study, however, how many IP_3_Rs participate of a global signal (a wave) via CICR coupling and how this number depends on the IP_3_R spatial distribution and the rate of Ca^2+^ removal. This study will help us interpret the experimental results. As explained in section 2, we mainly analyze the outcome of the simulation in terms of the number, *N*_*o*_, of IP_3_Rs that participate of an event, i.e., of a sequence or cascade of IP_3_R openings coupled via CICR. We also analyze the Ca^2+^ spatial distribution that would be observed if all the participating IP_3_Rs were simultaneously open by means of Equation (7).

**Table 3:** Values of the parameters used in the stochastic simulations.

**Parameter**	**Abbreviature**	**S1**	**S2**	**S3**	**S4**	**Unit**
Mean separation between clusters	dm	4	4	0.4	0.4	μ*m*
Mean number of clusters	λ_*N*_	25	25	2,500	2,500	*a*.*u*.
Mean number of IP_3_-bounded IP_3_Rs per cluster	*N*_*I*_*P*__3_*R*_	75	75	1	1	*a*.*u*.
Total number of IP_3_Rs	*N*_*T*_	2,095	2,099	2,383	2,567	*a*.*u*.
Ca^2+^ removal rate	δ_*Ca*_	200	20	200	20	*s*^−1^

**Figure 6:**
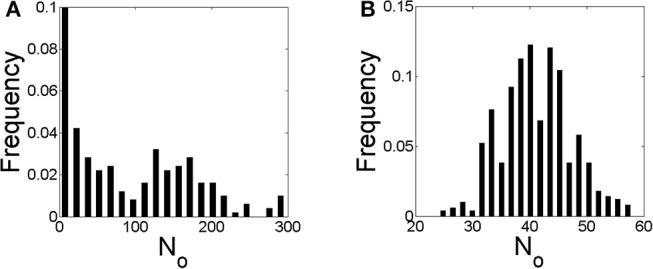
Distribution of the number of IP_3_Rs, *N*_*o*_, that participate of a global Ca^2+^ release event derived from stochastic simulations of the model described in section 2.4 but with a mean separation between clusters of 4μ*m* and $\delta_{Ca}=200s^{-1}$ **(A)** and $\delta_{Ca}=20s^{-1}$ **(B)**, i.e., conditions **S1** and **S2** of Table 3, respectively.

We show in Figure 6 the distribution of the number of IP_3_Rs that participate of each event, *N*_*o*_, obtained with numerical simulations performed using the parameters of cases **S1** (in Figure 6A) and **S2** (in Figure 6B) of Table 3. The main difference between the two simulations is the rate of Ca^2+^ removal which is $\delta_{Ca}=200s^{-1}$ in Figure 6A and $\delta_{Ca}=20s^{-1}$ in Figure 6B. The distribution of Figure 6A looks like the superposition of an exponential and a Gaussian, the latter centered at *N*_*o*_ ~ 150. In this figure, the first bin, *N*_*o*_ = 0, corresponds to the fraction of time steps of size *dt* = 0.05*s* during which there are no events. This fraction is 0.68 in this case. The scale of the figure has be chosen so that the distribution of events with *N*_*o*_ ≠ 0 is clearly observable. The distribution in Figure 6B looks like a Guassian centered at *N*_*o*_ ~ 40, a value that is similar to the mean, 〈*N*_*o*_〉 = 45.2. This value is much smaller than the one at which the distribution of Figure 6A has its local maximum, *N*_*o*_ ~ 150. In this case there are not time steps with no events.

**Figure 7:**
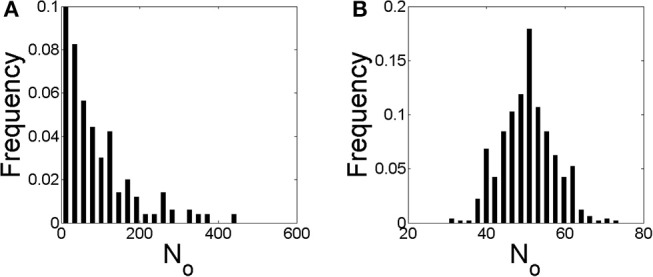
Similar to Figure 6 but with a mean separation between clusters of 0.4μ*m* and $\delta_{Ca}=200s^{-1}$ **(A)** and $\delta_{Ca}=20s^{-1}$ **(B)**, i.e., conditions **S3** and **S4** of Table 3, respectively.

We show in Figure 7 similar figures to those of Figure 6 but derived from simulations for which the IP_3_Rs were more evenly distributed in space (case **S3** of Table 3 in Figure 7A and case **S4** in Figure 7B). The *N*_*o*_ distribution of Figure 7A is approximately exponential and shows (rare) events with larger values of *N*_*o*_ than those of Figure 6A. It does not have a local maximum as the one in Figure 6A. The fraction of time with no events is 0.65, similar to that of Figure 6A. The *N*_*o*_ distribution of Figure 7B is Gaussian like, as in Figure 6B, with a maximum at *N*_*o*_ ~ 50, a very similar value to the mean, 〈*N*_*o*_〉 = 55.5. The ratio between the mean values of Figures 6B, 7B (〈*N*_*o*_〉 = 45.2 and 〈*N*_*o*_〉 = 55.5, respectively) is similar to the ratio between the total number of IP_3_Rs of both simulations (*N*_*T*_ = 2, 099 for case **S2** and *N*_*T*_ = 2, 567 for **S4**). Also for the case of Figure 7B there are no time steps with no events.

**Figure 8:**
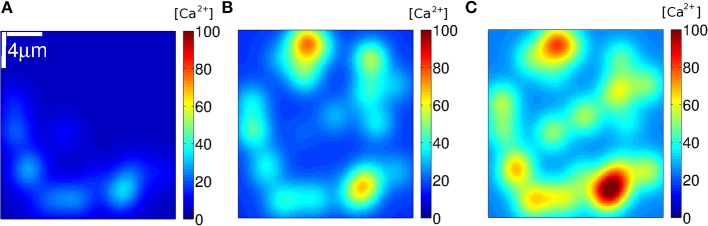
[Ca^2+^] distribution of events in three instants **(A–C)** of stochastic simulations with a mean separation between clusters of 4μ*m* and $\delta_{Ca}=200s^{-1}$ (condition **S1**). Example **(A)** corresponds to *N*_*o*_ = 68, **(B)** to 203 and **(C)** to 255.

**Figure 9:**
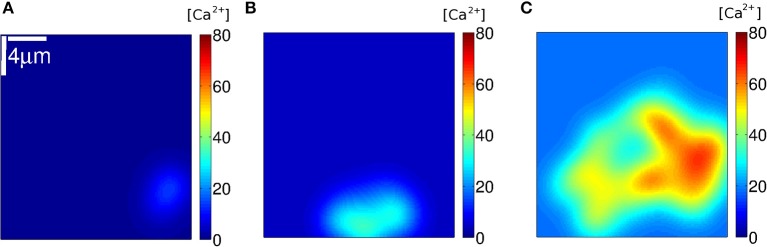
[Ca^2+^] distribution of events in three instants **(A–C)** of stochastic simulations with a mean separation between clusters of 0.4 μ*m* and $\delta_{Ca}=200s^{-1}$ (condition **S3**). Example **(A)** corresponds to *N*_*o*_ = 9, **(B)** to 49 and **(C)** to 215.

**Figure 10:**
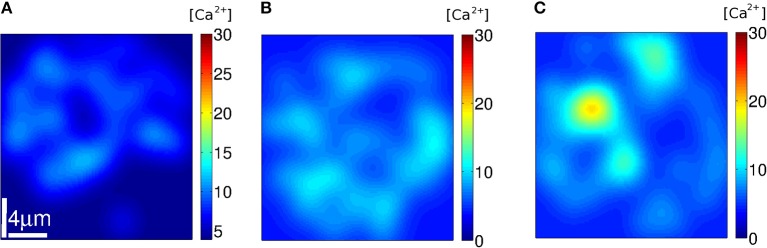
[Ca^2+^] distribution of events in three instants **(A–C)** of stochastic simulations with a mean separation between clusters of 0.4μ*m* and $\delta_{Ca}=20s^{-1}$ (condition **S4**). Example **(A)** corresponds to *N*_*o*_ = 33, **(B)** to 44 and **(C)** to 48.

We used Equation (7) to estimate how the [Ca^2+^] distribution would look like during signals with the number and location of the IP_3_Rs that participated of an event according to the simulations. We show plots of the distributions for three such events in Figures 8–10. The example of Figure 8 was drawn from the simulation of case **1**, i.e., a situation where the IP_3_Rs were spatially clustered and the Ca^2+^ removal rate was high. The example of Figure 9 was drawn from the simulation of case **3**, i.e., a situation where the IP_3_Rs were more uniformly distributed in space and the Ca^2+^ removal rate was high. The example of Figure 10 was drawn from the simulation of case **4**, i.e., a situation with uniformly distributed IP_3_Rs and low Ca^2+^ removal rate.

## Discussion and Conclusions

Ca^2+^ signals are ubiquitous. Their versatility relies on the variety of spatio-temporal distributions that the intracellular Ca^2+^ concentration can display. These distributions are the result of the interplay between geometry (the spatial location of the components that participate of the signals) and dynamics (determined by the rates of Ca^2+^ transport, Ca^2+^ buffering, Ca^2+^ removal, and Ca^2+^ release into the cytosol). The motivation of this paper was to study this interplay for signals in which Ca^2+^ is released from the ER through IP_3_Rs. More specifically, we sought to determine the relative role of the non-uniform IP_3_R spatial distribution on the resulting signal.

The non-uniform distribution of IP_3_Rs in many cell types plays a major role for the type of signals that are elicited (Keizer et al., 1998; Dawson et al., 1999; Sun et al., 2011; Ullah et al., 2014). In particular, the spatial range of the signals largely depends on whether the Ca^2+^ released from one IP_3_R cluster can induce the opening of IP_3_Rs in nearby clusters. The inability to induce this sequence of openings results in propagation failure (Pearson and Ponce-Dawson, 1998). It is also exploited experimentally when slow Ca^2+^ buffers are used to disrupt CICR between clusters and elicit only local signals, i.e., puffs (Dargan et al., 2004; Piegari et al., 2015). It is known that, as the oocyte gets transformed into egg, there is a reconfiguration of the ER that affects the IP_3_R spatial distribution (Terasaki et al., 2001). The differences in the Ca^2+^ signals observed in oocytes and eggs have been attributed as being partly due to this change in the spatial IP_3_R distribution (Sun et al., 2011; Ullah et al., 2014). The changes that occur with maturation thus provide an ideal setting to study the effect of geometry on the resulting signals. In this paper we relied on the changes that occur with maturation to ponder the relative role, on IP_3_R-mediated Ca^2+^ signals, of the spatial IP_3_R-distribution and of the other processes that modulate the Ca^2+^ dynamics.

The aim of the experiments was to determine whether the signals elicited in immature and in artificially matured *X. laevis* oocytes were more or less spatially uniform in one or the other setting when subject to the same pattern of IP_3_ photo-release. In the case of the oocytes, we observed several spotlights of Ca^2+^ release “turned on” before the signal became a propagating wave (Figures 2A–C and Supplementary Video 1). We also observed them between the end of the first UV flash and the beginning of the second. These localized events were unobservable in the case of the eggs for which the signals looked much more continuous (Figure 3 and Supplementary Video 2). Similar behaviors were observed in the experiments performed with the other UV flash durations probed (data not shown). The differences in the spatial distribution of the signals of Figures 2, 3 were also apparent in Figures 4C,D, where we showed the time-course of the mean, *F*_*m*_(*t*) (Equation 2), and of *F*_*m*_(*t*)±σ_*F*_(*t*) with, σ_*F*_(*t*), the deviation (Equation 3) computed over the 250 × 250-pixel regions of the images of Figures 2, 3, respectively, that had similar initial values of $\bar{F}$ before the UV flash [*n* = 20 regions with $\bar{F}\in \left(2.6,3.8\right)a.u.$ for the oocyte and *n* = 21 regions with $\bar{F}\in \left(1.4,2\right)a.u.$ for the egg]. We observed that the deviation was larger in the oocyte than in the egg which means that the Ca^2+^ concentration difference between regions (and, therefore, the Ca^2+^ gradient) were larger in the former. Given that the non-uniformity could pre-exist the UV flash, we plotted in the insets the ratio, $\sigma_{F}\left(t\right)/\sigma_{{\bar{F}}_{b}}$, with $\sigma_{{\bar{F}}_{b}}$, the deviation before the first UV flash was delivered. There we observed that the ratio was also smaller in the case of the egg than in the oocyte. We also compared the time course of the fluorescence, $\bar{F}$, in the two contiguous regions indicated with white boxes in Figures 2F, 3F which we plotted, respectively, in Figures 4A,B. From these figures we computed the rise time, *t*_*r*_, of $\bar{F}$ and the ratio between its maximum value and the value it had immediately before each UV flash. The ratio differed by a factor ~2–3 between Regions 1 and 2 of Figure 2F while these differences were less than 1% for Regions 1 and 2 of Figure 3F. The values of *t*_*r*_, on the other hand, were practically the same in the two regions compared in both cases. The value, *t*_*r*_, itself was ~ 6 times smaller while the maximum fluorescence attained was ~ 4.5 larger in the oocyte than in the egg (see Figure 4). The fact that the signals seemed to be more spatially uniform in eggs that in oocytes was further reflected in Tables 1, 2 where we listed the mean and standard deviation of the characteristic times derived from the fits to the decaying part of the fluorescence time courses, ${\bar{F}}_{k}$, obtained in the subregions of the images with similar basal fluorescence levels (Equation 1) of all the experiments performed in oocytes and in eggs that we reported in this paper. The ratio between the standard deviation and the mean of the obtained parameters varied between ~ 0.1 and 0.7 in the case of the best fits (bi-exponential) of the oocyte, while it was ~ 0.08 or less for the most of the best fits of the egg.

The recovery of the spatially averaged fluorescence observed in Figures 2, 3 once the UV flash was turned off also presented differences between the oocyte and the egg that could be attributed to more or less spatially uniform Ca^2+^ distributions. As illustrated in Figures 4C,D, while the deviation at the time at which *F*_*m*_ was maximum increased by a similar factor with respect to the basal level in the oocyte and in the egg (it was 1.4 larger than before the UV flash in the oocyte and 1.3 larger in the case of the egg), the subsequent behavior was different. In particular, the ratio between the standard deviation and *F*_*m*_ at the time of the maximum of *F*_*m*_ and at 2.8 s after the UV flash had been turned off was, respectively, 0.17 and 0.09 for the oocyte and 0.13 and 0.14 for the egg. The faster decrease of the deviation in the oocyte implies that, once the photo-release of IP_3_ (and presumably, of Ca^2+^) ceases, the Ca^2+^ concentration gets more uniform in the oocyte than in the egg. This could be explained in terms of diffusion. The spatially less uniform Ca^2+^ distribution in the oocyte would lead to larger concentration gradients that would then dissipate fast due to diffusion. Diffusion would not be as efficient to remove Ca^2+^ away from the observed region in the egg due to the more uniform distribution of the ions. The occurrence of this fast clearance that we associate to diffusion is also reflected in the different ways in which *F*_*m*_ decays with time. Namely, the decay time of *F*_*m*_ in the oocyte was best fitted by a bi-exponential in all the experiments as shown in Table 1, with a fast component that we associate to this clearance due to diffusion. The decay in the case of the egg, on the other hand, did not have this fast component (Table 2).

The results discussed so far give evidence that the [Ca^2+^] is more spatially uniform during the signals evoked in the eggs than in the oocytes. We could also observe other differences in the experiments, some of which reinforce this conclusion. In particular, we observed differences in the growth of the fluorescence between the egg and the oocyte as illustrated in Figures 4, 5. We observed in Figures 4A,C (which correspond to the oocyte) that both ${\bar{F}}_{k}$ and the mean, *F*_*m*_, showed a marked peak that occurred approximately at the same time as the localized spots observed in Figure 2 (~ 12*s*), while they increased much more slowly and reached smaller values in the egg (Figures 4B,D). For the egg, the maxima of ${\bar{F}}_{k}$ and *F*_*m*_ were attained approximately when the UV flash was turned off. The decrease of ${\bar{F}}_{k}$ in the oocyte while the UV flash was still on was more pronounced in the region with the higher fluorescence amplitude of the two illustrated in Figure 4A (Region 1 of Figure 2F). In regions with lower fluorescence values, $\bar{F}$ remained relatively constant at its (local) maximum during most of the UV illumination time (data not shown). This type of plateau was also observed in regions of the egg with relatively large $\bar{F}$ values. We also computed the rise time, *t*_*r*_, of *F*_*m*_ for Figures 4C,D obtaining a value that was 4−5 times smaller in the oocyte than in the egg. The ratio between the maximum of *F*_*m*_ and its value before the UV flash was also 2.2 larger in the oocyte than in the egg. These observations were replicated for some of the examples of Figure 5. We observed in this figure that, both in eggs and oocytes, $\bar{F}$ reached its maximum value at the time at which the UV flash was turned off for the shortest flash duration probed. This behavior persisted for the 28 s duration flashes in the case of the eggs but not of the oocytes. Finally, the peak occurred in oocytes and eggs before the flash had been turned off for the longest flash duration.

The observations discussed so far are compatible with having a faster growth of the global Ca^2+^ signal observed in the oocyte of Figure 2 than in the egg of Figure 3. The difference in the initial rise of the fluorescence in the oocyte and the egg can be associated to different IP_3_R spatial distributions. Namely, very packed *intra-cluster* IP_3_R distributions lead to a more efficient CICR which results in higher Ca^2+^ elevations and, at the same time, contributes to a faster propagation of the signal between clusters. Figure 4C also showed that the maximum mean fluorescence attained was smaller for the second pulse compared to the first one in the case of the oocyte while this difference was unobservable in the example of the egg (Figure 4D). The amplitude difference occurred in the oocyte although the mean fluorescence at the beginning of the second pulse was larger than at the beginning of the first one (3.1 to 3.3*a*.*u*.). We can interpret this lower elevation as being due to the existence of a subset of IP_3_Rs that, when the second flash was applied, still remained inactive after the signal evoked by the first flash. The absence of this difference in the egg indicates that the pool of activatable IP_3_Rs was approximately the same for both flashes in this example. However, we did not obtain this same behavior in other experiments performed in eggs. Although the experiments performed with varying durations of the UV flash showed smaller fluorescence amplitudes for eggs than oocytes (Figure 5), they also showed that, in most cases, both for oocytes and eggs, the amplitude of the second peak was smaller than that of the first one and that the difference increased when we increased the UV flash duration.

We can interpret some of these observations in terms of the IP_3_R kinetics and the IP_3_R spatial distribution. In particular, the regions where the largest fluorescence amplitudes were observed in each case can be associated to regions where the number of simultaneously open IP_3_Rs was largest. The fact that the fluorescence increased faster in the regions where it reached the largest amplitudes seems to indicate that IP_3_Rs are closer together in those regions. Given that, for the same UV flash duration, the maximum values of $\bar{F}$ were larger for oocytes than for eggs can be reflecting that IP_3_Rs are more tightly packed in oocytes than in eggs. The observations that, if the amplitude of the first peak is large enough, the second peak tends to be smaller than the first one; that, for the same UV flash duration, the amplitude difference between the two peaks is larger for the oocytes than for the eggs and that this difference increases with the amplitude of the first peak can be interpreted in terms of the inhibition of IP_3_Rs after they participate of a signal. In particular, they indicate that, if the number of IP_3_Rs that participated of the signal evoked by the first UV flash is too large, then many of them remain inhibited when the new flash is delivered. The smaller amplitude observed in eggs could be due to a smaller number of IP_3_Rs, to a less efficient CICR coupling due to the different IP_3_R spatial distribution or could be an indication that after a first “sweep” where many IP_3_Rs take part in a signal, the system starts to approach a new stable state as we observe with the numerical simulations in which the rate of Ca^2+^ removal is not large enough. The existence of a first “sweep” involving many more open IP_3_Rs than immediately afterwards is apparent in the images obtained in eggs illustrated in the inset of Figure 4D and in Figures 5D–F. The differences observed between eggs and oocytes in the way that $\bar{F}$ behaves while the flash is on can also be interpreted in terms of differences in the IP_3_R spatial distribution. The fact that the fluorescence can remain constant or decay while IP_3_ is being photorelased (at a slower pace than while the UV flash is off) indicates that there is still Ca^2+^ release during that time but that this release is unable to overcome the processes that remove Ca^2+^ from the observed region. Given that, for the same amount of IP_3_ released, it takes longer for $\bar{F}$ to reach a plateau in eggs than in oocytes points to a more efficient recruitment of IP_3_Rs in the latter. After having been open, IP_3_Rs typically enter an inhibited state. Thus, the more efficient IP_3_R recruitment in oocytes implies that IP_3_Rs become inhibited faster in these cells than in eggs which could explain the faster decay of $\bar{F}$ while the UV flash is still on. This more efficient recruitment can also explain the observation that the second pulse of Ca^2+^ release has usually a smaller amplitude than the first one in oocytes, even for short durations of the IP_3_ release and that this occurs for eggs for long enough UV flashes. An efficient recruitment would imply that the number of IP_3_Rs that become open during the first round of IP_3_ release is so large that a significant amount of them is still inhibited when the second UV flash is shone which, in turn, would lead to a lower amplitude Ca^2+^ pulse. Now, the decay of $\bar{F}$ while the UV flash is on or the fact that the amplitude of the second flash is smaller than the first one when the first amplitude could also be due to the cell “running out” of IP_3_. In order to discard this possibility all the experiments were repeated for a new round 10 min after the second UV flash obtaining larger amplitudes in the third flash than in the second one (data not shown).

The way the fluorescence decayed in oocytes and eggs once the UV flash was turned off not only differed in the existence of a fast component that we only observed in the former and that we attributed to diffusion, but also differed in the characteristic time-scales as reflected in Tables 1, 2. Comparing the results of the best fit in each case we conclude that the decay times were smaller for the oocyte than for the egg. As the Ca^2+^ gradient dissipates, the role of pumps (and buffers) is more important. Thus, we can expect that the slow component of the decay in oocytes and perhaps all of the decay in the case of eggs is dominated by this process. Given the smaller [Ca^2+^] that we observe in eggs (see Figures 4, 5), on the other hand, we could expect the removal rate to be [Ca^2+^]-dependent (i.e., that the pumps are not saturated). However, the good linear fits that we obtain (see Table 2) seems to indicate that the pumps are saturated even for the small [Ca^2+^] that we obtain in many of the experiments performed in eggs. The hypothesis that the pumps are saturated is compatible with the results presented in El-Jouni et al. (2005), where they show that, in eggs, the Plasma Membrane Ca^2+^ ATPase (PMCA) is completely internalized so that Ca^2+^ cannot be removed to the extracellular medium. This limitation to remove cytosolic Ca^2+^ together with the permanent loss of Ca^2+^ from the ER through IP_3_Rs could explain why Ca^2+^ could remain at relatively large concentrations for a longer time in eggs than in oocytes (El-Jouni et al., 2005). The efflux of Ca^2+^ from mitochondria is also apparent in some of the experiments performed in eggs. Particularly in Figure 5D we can observe the two-phase decay obtained in simulations of the model of Falcke et al. (1999) for cases with high mitochondrial uptake. As discussed in Falcke et al. (1999), this results in a prolonged elevation of cytosolic Ca^2+^. In the experiments performed for different durations of the UV flash (Figure 5) the decay of the fluorescence was best fitted with a mono-exponential for the 56 s flash duration for which we obtained a decaying rate (83±1.4) s immediately after the first UV pulse. If we associate the linear decay of the fluorescence to the removal of Ca^2+^ due to saturated pumps, we can explain the change to a mono-exponential decay with increasing [Ca^2+^] assuming that there are low affinity buffers that participate of the Ca^2+^ clearance only when [Ca^2+^] is large enough (Figure 5F).

Our experiments thus showed differences in the spatio-temporal distribution of the [Ca^2+^] in eggs and oocytes that can be interpreted in terms of different spatial organizations of the IP_3_Rs, particularly, in terms of a more spread and spatially uniform IP_3_R distribution in eggs than in oocytes. The observation of marked IP_3_Rs in eggs and oocytes showed patches in the former that could be the origin of this apparent more uniform distribution, but patches do not comprise the whole cell (Sun et al., 2011). Furthermore, the simulations of Ullah et al. (2014) assumed more separated IP_3_R clusters in eggs than in oocytes. In any case, a more spatially uniform IP_3_R distribution in eggs than in oocytes is not necessarily the only way to explain the differences that we observed in [Ca^2+^] as we discuss in what follows. The experiments also showed other differences that could be attributed to the changes that are known to occur in the mechanisms of Ca^2+^ removal with maturation (El-Jouni et al., 2005). In order to study the interplay between these various processes and to further interpret our experimental observations we produced numerical simulations of an extended version of the simple model introduced in Lopez et al. (2012). The model does not consider a time-dependent [IP_3_] as in the experiments. In any case it can serve to describe what we observed experimentally during the duration of the UV flashes. In any case, given that the model includes the other processes that modulate the signals it thus allows us to study the ways in which these various processes compete to produce different outcomes. The model has other limitations. It does not consider the depletion of the Ca^2+^ stores due to the IP_3_R-mediated Ca^2+^ release. This might not be that important given that, as analyzed in Lopez and Dawson (2016), luminal Ca^2+^ is usually readily available for IP_3_R-mediated release in oocytes. It does not describe either the time during which the signal propagates or the time over which diffusion acts to make [Ca^2+^] uniform once the release of Ca^2+^ stops. It is implicit in the latter that the spatial homogenization of [Ca^2+^] occurs fast enough so that it is unlikely that a new signal will occur before [Ca^2+^] is more or less uniform again. The model then serves to study how many IP_3_Rs can be coupled via CICR and participate of a signal and how this depends on the rate at which Ca^2+^ is removed by pumps and buffers. With the simulations we analyzed the number, *N*_*o*_, of IP_3_Rs that participated of a given release event (a propagating signal or *cascade*) due to CICR. In particular, we studied how the distribution of *N*_*o*_ values varied depending on the IP_3_R spatial distribution and the rate of Ca^2+^ removal for a fixed mean value of IP_3_-bound IP_3_Rs (see Table 3). We found three types of *N*_*o*_ distributions: one that looked exponential (Figure 7A), another that looked Gaussian about a mean 〈*N*_*o*_〉 ≠ 0 (Figures 6B, 7B) and one that was intermediate between the two, with an exponential dependence for small values of *N*_*o*_ and a “bump” around a mean away from *N*_*o*_ = 0 (Figure 6A) that might be due to a border effect. We obtained the Gaussian (and the “mixed”) *N*_*o*_ distribution both when IP_3_Rs were more uniformly distributed in space (Figure 7) and when they were clustered (Figure 7). The transition between the *N*_*o*_ distribution types seemed to be mostly determined by the rate of Ca^2+^ removal.

For the largest Ca^2+^ removal rate that we tried, the distribution always had an exponential part either for all *N*_*o*_ values (Figure 7A) or just for the smallest ones (Figure 6A). The parameters of the simulations of Figures 6A, 7A (cases **S1** and **S3** of Table 3, respectively) differed in the IP_3_R spatial distribution, which was more uniform in case **S3**. In both types of simulations, the fraction of time with no events was similar (~ 0.65−0.68 of the 50 s total simulation time). For the case in which the rate of Ca^2+^ removal was low, the distribution was approximately symmetric around a mean value, 〈*N*_*o*_〉 ~ 0.02*N*_*T*_ with *N*_*T*_ the total number of IP_3_Rs with IP_3_ bound. This happened both when the spatial distribution of IP_3_Rs was approximately uniform (case **S4**, Figure 7B) and when it was clustered (case **S2**, Figure 6B). The third behavior occurred for the case with high Ca^2+^ removal rate and clustered IP_3_Rs (case **S1**, Figure 6A). In this case there is an apparent local maximum around *N*_*o*_ ~ 150.

We subsequently analyzed how the Ca^2+^ spatial distribution would look like if all IP_3_Rs that participated of a cascade were simultaneously open. This analysis allowed us to interpret the *N*_*o*_ probability distributions and gave additional information. We illustrated the Ca^2+^ spatial distribution during 3 events obtained with: clustered IP_3_Rs and high Ca^2+^ removal rate (case **1**, Figure 8); more uniformly distributed IP_3_Rs and high Ca^2+^ removal rate (case **3**, Figure 9) and more uniformly distributed IP_3_Rs and low Ca^2+^ removal rate (case **4**, Figure 10). The examples of Figure 8 correspond, respectively, to events with *N*_*o*_ = 68, 203, and 255; those of Figure 9 to *N*_*o*_ = 9, 49, and 215 and those of Figure 10 to *N*_*o*_ = 38, 44, and 48. The examples of Figures 8, 9 correspond to values of *N*_*o*_ in different regions of the corresponding probability distributions (Figures 6A, 7A, respectively) while those of Figure 10 are close to the mean of the distribution of Figure 7B). We observed in Figure 8 that, in all cases, the Ca^2+^ spatial distribution was not uniform. The main difference between large or small *N*_*o*_ was the spatial localization of the event in the latter as opposed to a more spread signal in the former. But still, in all cases, the sites of Ca^2+^ release could be identified due to the relatively larger Ca^2+^ concentration around them. The spatial Ca^2+^ distribution, on the other hand, was pretty uniform in all the cases illustrated in Figure 9. Even though there is a local peak in Figure 9C, it is important to notice the different scales used in Figures 8, 9 which enlarges the concentration differences in the latter. The maximum [Ca^2+^] value in the example of Figure 8 (*N*_*o*_ = 68) was higher than in all the examples of Figure 9 (*N*_*o*_ = 38, *N*_*o*_ = 44, and *N*_*o*_ = 48) but had a much more localized Ca^2+^ spatial distribution. The maximum values of Figures 8B,C were higher than those in Figure 9. The maximum [Ca^2+^] values in the examples of Figure 10 were intermediate between those of the other two figures. The Ca^2+^ distribution looked locally more uniform in Figure 10 than in Figure 8, due to the more uniform underlying IP_3_R distribution, bur did not spread over the whole domain as observed in the examples of Figures 8B,C, 9B,C. This difference in the [Ca^2+^] spatial distribution was apparent even for events with similar values of *N*_*o*_ (e.g., Figures 8B, 10C for which *N*_*o*_ = 203 and 215, respectively).

The Ca^2+^ concentration before the start of the cascade was quite similar in the Figure 10 which correspond to case **S4**, i.e., the conditions for which the *N*_*o*_ distribution was Gaussian-like about the mean 〈*N*_*o*_〉 = 56 (Figure 7B). This pre-cascade concentration varied between ~ 6 and 25μ*M* in Figure 8 and between 0.9 and 18μ*M* in Figure 9, the two cases for which the *N*_*o*_ distribution had an exponential like behavior either for the smallest (case **S1**, Figure 6A) or for all *N*_*o*_ values (case **S3**, Figure 7A). The signals propagate via CICR and this mechanism is more or less effective depending on the [Ca^2+^] prior to the opening of the first channel, on the number of active IP_3_Rs in each cluster and on the distance between clusters with active channels. On the other hand, the probability of opening the first channel at a certain time, *t*, depends on [Ca^2+^] and the number of active IP_3_-bound IP_3_Rs at that time. Both the [Ca^2+^] value and the number of active IP_3_Rs vary with time. If [Ca^2+^] is too low, the possibility of coupling different clusters via CICR is mainly limited by the distance between the clusters and the amount of Ca^2+^ that can be released from each of them. In those cases (e.g., the examples of Figures 8A, 9A) it is most likely that a few clusters will be coupled via CICR. This, in turn, limits the number of IP_3_Rs that participate of the cascade to be only a few. This scenario is likely to occur quite often if Ca^2+^ removal occurs at a fast pace as in the situations of Figures 8, 9. In the case of fast Ca^2+^ removal, only in the few instances in which there is enough Ca^2+^ in the medium and a sufficiently large number of uninhibited IP_3_Rs at the start of the event the corresponding cascade will involve the participation of many IP_3_Rs, like in Figures 8C, 9C. It is important to note that for these subfigures both [Ca^2+^] and *N*_*o*_ were the largest of the three examples of Figures 8, 9, respectively. This difference in how likely it is to have events with more or fewer IP_3_Rs could explain the exponential like behavior of the *N*_*o*_ distributions of Figures 6A, 7A. If Ca^2+^ removal does not occur fast enough, once an IP_3_R becomes uninhibited it is very likely that it will become open soon after that. Given the relatively “large” (and uniform) [Ca^2+^], then, distant uninhibited IP_3_-bound IP_3_Rs will be coupled via CICR. In this way, the number, *N*_*o*_, of IP_3_Rs that participate of an event will be mostly determined by the number of simultaneously active IP_3_Rs, no matter how far away from one another they are (within certain limits). In this case, spatial heterogeneities do not play much of a role: the slow Ca^2+^ removal smears out inhomogeneities and couples relatively distant regions. If there are sufficiently many IP_3_Rs in the whole system, it is likely that several IP_3_Rs will be simultaneously uninhibited. Thus, the signal will then spread throughout the domain. In summary, we associate the exponential part of the *N*_*o*_ distribution to those cascades which occurrence is limited by CICR, i.e., by the IP_3_Rs binding the Ca^2+^ that is released by other IP_3_Rs. On the other hand, we interpret the Gaussian-like distribution as being the consequence of a long-distance coupling where space inhomogeneities are less visible.

**Figure 11:**
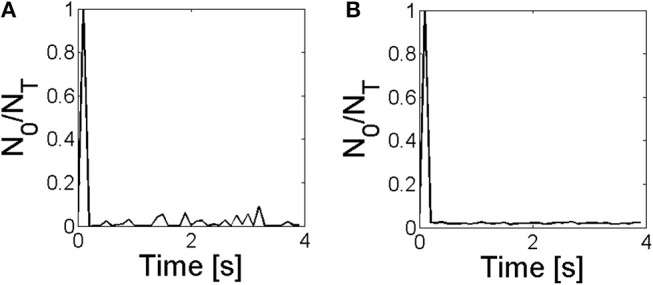
Fraction of IP_3_Rs that participate of a global Ca^2+^ release event, *N*_*o*_/*N*_*T*_, derived from stochastic simulations of the model described in section 2.4 with a mean separation between clusters of 0.4μ*m* and $\delta_{Ca}=200s^{-1}$ **(A)** and $\delta_{Ca}=20s^{-1}$ **(B)**, it is conditions **S3** and **S4** of Table 3, respectively.

The Gaussian like event size distribution (Figures 6B, 7B) is indicative that the system bifurcates, when the Ca^2+^ removal rate is decreased, to a bistable situation with one fixed point corresponding to basal Ca^2+^ and no IP_3_Rs open and the other corresponding to a higher Ca^2+^ level and *N*_*o*_ open IP_3_Rs with *N*_*o*_ equal to the value at which the Gaussian has its maximum. We recall that such a situation could be maintained provided that the turn-over time of luminal Ca^2+^ was fast enough to guarantee Ca^2+^ release every time there is an open IP_3_R. So, the actual situation could be one in which the release starts eventually to decline. In any case, we do not want to analyze this possibility here. The exponential like distribution (Figures 6A, 7A) is indicative that the system is excitable, so that most often events are evoked that do not spread much in space (small *N*_*o*_) while less often events are evoked that involve the opening of many IP_3_Rs (large *N*_*o*_). The latter sends most of the IP_3_Rs of the system into an inhibited state which delays the occurrence or reduces the number of participating IP_3_Rs of the subsequent event (as in Fraiman et al., 2006). In the bistable case, on the other hand, after a very short transient, the number of IP_3_Rs that participate of each event fluctuates around a mean that is only a fraction of the total number of IP_3_Rs of the system. Thus, IP_3_Rs do not enter the inhibited state simultaneously as they do after the largest events of the excitable case. This means that active IP_3_Rs are readily available to become open at any given time which guarantees the spatial spread of the Ca^2+^ signal. This could explain the observations of Figure 4D, in which a very large deviation is observed for a very brief time immediately after the UV flash is turned on that then settles to a smaller and relatively constant value and the occurrence of two peaks of more less the same height which means similar numbers of simultaneously open IP_3_Rs in both. This is in fact observed in the simulations as illustrated in the Figure 11 where we show that there are almost no time steps without events in the simulations with small Ca^2+^ removal rate and that, after a transient, the fraction of IP_3_Rs that participate of the events fluctuates very little around the mean that can be associated to the high [Ca^2+^] fixed point.

The results of the experiments and of the numerical simulations presented in this paper show that even though the non-uniform distribution of IP_3_Rs is relevant for the different types of signals observed in eggs and oocytes, the rate of Ca^2+^ removal is key since it can smear out spatial inhomogeneities. All our results indicate that Ca^2+^ removal due to pumps and buffering occurs much more slowly in eggs than in oocytes. This is consistent with previous observations. As we have already mentioned, the PMCA is completely internalized in eggs (El-Jouni et al., 2005). The difference between the velocity of the fertilization wave in eggs and of the saltatory waves observed in oocytes, on the other hand, could be explained in Dawson et al. (1999) with a Ca^2+^ diffusion coefficient that was twice as large in the former case. This larger effective diffusion coefficient is, in turn, compatible with less effective Ca^2+^ buffers. The results of Figure 5 indicate that the buffers that act in eggs are of relatively low affinity. As observed in a variety of papers (Miller et al., 1993; Creton et al., 1998), the addition of fast Ca^2+^ buffers disrupts the steps that are necessary for development to advance. In fact, [Ca^2+^] needs to reach relatively high values for the steps that follow fertilization to take place. Simulations of the fertilization wave in *X. laevis* oocytes (Wagner et al., 1998), in turn, supported the hypothesis that the physiological state of the mature egg was bistable. In those simulations the transition from an oscillatory regime in immature oocytes to a bistable one in eggs was explained assuming that the rate of Ca^2+^ release increased with maturation. A similar transition could be explained, however, for a decreasing rate of Ca^2+^ removal (to which the efflux of Ca^2+^ from mitochondria observed in eggs could also contribute; Falcke et al., 1999). Our observations would favor this last hypothesis.

## Data Availability

All datasets generated for this study are included in the manuscript and/or the Supplementary Files.

## Author Contributions

EP and CV performed the experiments. EP and SP performed the simulations. EP, CV, and SP analyzed the experiments and simulations and wrote the paper. SP conceived the work.

### Conflict of Interest Statement

The authors declare that the research was conducted in the absence of any commercial or financial relationships that could be construed as a potential conflict of interest.

## Supplementary Material

Supplementary Video 1 Video of a Ca^2+^ signal evoked in an immature oocyte. Frames of 250 × 250 pixels acquired aevery 0.56 s. Warmer colors correspond to increasing fluorescence values in arbitrary units (*a*.*u*.). The UV illumination (used to uncage the IP_3_) was on between *t* = 11.2 s and *t* = 28 s and between *t* = 173.6 s and *t* = 190.4 s.

Supplementary Video 2 Video of a Ca^2+^ signal evoked in an egg. Similar to Supplementary Video 1 but for an experiment performed in an egg. In this case the UV illumination was on between *t* = 11.2 s and *t* = 28 s and between *t* = 112 s and *t* = 128.8 s.
